# Creating Refined Datasets for Better Chaos Detection

**DOI:** 10.3390/s25030796

**Published:** 2025-01-28

**Authors:** Dariusz R. Augustyn, Katarzyna Harężlak, Agnieszka Szczęsna, Henryk Josiński, Paweł Kasprowski, Adam Świtoński

**Affiliations:** 1 Department of Applied Informatics, Faculty of Automatic Control, Electronics and Computer Science, Silesian University of Technology, Akademicka 16, 44-100 Gliwice, Poland; katarzyna.harezlak@polsl.pl (K.H.); pawel.kasprowski@polsl.pl (P.K.); 2 Department of Computer Graphics, Vision and Digital Systems, Faculty of Automatic Control, Electronics and Computer Science, Silesian University of Technology, Akademicka 16, 44-100 Gliwice, Poland; agnieszka.szczesna@polsl.pl (A.S.); henryk.josinski@polsl.pl (H.J.); adam.switonski@polsl.pl (A.Ś.)

**Keywords:** time series, phase portrait, clustering, classification, chaos detection

## Abstract

In recent years, the analysis of signal properties (especially biomedical signals) has become an important research direction. One interesting feature of signals is their potential to be chaotic. This article concerns the issues of classification of real signals or synthetic ones in the context of detecting chaotic properties. In previous works, datasets of synthetic signals were created based on well-known chaotic and non-chaotic dynamical systems. They were published and used to train classifiers. This paper extends the previous studies and proposes a method for obtaining/extracting signals to force classifiers to learn to detect chaos. The proposed method allows the generation of groups of signals with similar initial conditions. The property of chaotic dynamical systems was used here, which consists of the strong dependence of the signal courses on a small change in the initial conditions. This method is based on reconstructing multidimensional phase space and data clustering. An additional goal of the work is to create referential datasets with so-called refined signals using the described method and to make them publicly available. The usefulness of the new datasets was confirmed during a simple experiment with the usage of the LSTM neural network.

## 1. Introduction

The analysis of biomedical signals has attracted increased interest during the last decade. This interest stems from technological development that allows the measurement of various physiological parameters, among which electrocardiograms, human gait kinematic data, electroencephalograms, photoplethysmograms, and eye movements (video-oculography and electrooculography) are notable. The exploration of these signals is aimed at understanding and modeling their nature, which is of great importance, especially for medical diagnosis. Such investigations may be found inter alia in [1,2] for electrocardiograms, ref. [3] for electroencephalograms, ref. [4] for photoplethysmograms, refs. [5,6] for gait, and [7] for eye movement.

Based on the recorded biomedical signal time series, previous studies revealed that natural physical phenomena are interpreted as nonlinear dynamical systems [8,9] and can exhibit chaotic or non-chaotic characteristics. Therefore, effort was put into determining these systems’ behavior patterns to differentiate various states and events [10,11,12]. This kind of exploration utilizes nonlinear time series analysis, including the time-delay reconstructed trajectory, correlation dimension, and largest Lyapunov exponent. However, the obtained results do not always confirm the same findings, as was the case for the works [13,14]. Thus, other approaches are being searched for.

Recent techniques in this field use machine learning methods. In [15], the authors applied such an approach to classify univariate time series generated by discrete and continuous dynamical systems with known characteristics. They obtained satisfactory results and suggested that deep learning methods can also differentiate real-life data into chaotic and non-chaotic signals. A similar work was presented in [16], where the authors focused on classifying three chaotic signals generated by the well-known Lorenz, Chen, and Rössler dynamical systems using different step intervals, initial values, system parameters, and time intervals. Four machine learning methods were utilized—Support Vector Machines, Naive Bayes, k-Nearest Neighbors, and Decision Tree—among which kNN turned out to be the most effective, with accuracy reaching 99%.

Because a limited group of systems were considered in those studies, an extended set of signals was developed [17] to provide more expansive possibilities for deeper signal exploration. The dataset consists of 13 dynamical systems (five chaotic and eight non-chaotic) (https://draugustyn.gitlab.io/signal-data/ (accessed on 16 January 2025)) of the first, second, or third order. A total of 1000 files, each containing 1000 samples, were provided for each system. They are publicly available at the Figshare project titled “Datasets for learning of unknown characteristics of dynamical systems” (https://figshare.com/projects/Datasets_for_learning_of_unknown_characteristics_of_dynamical_systems/140275 (accessed on 16 January 2025)). Several experiments were conducted to verify the usability of the developed datasets [4,18]. The wavelet, simple LSTM (long short-term memory), and CNN (convolutional neural network) models for signal classification were tested. The results revealed that simple architectures were sufficient to achieve satisfactory performance, reaching an accuracy of 98%.

The study presented in this paper focused on further improvements in training models that differentiate chaotic and non-chaotic signals. Signal time series clustering is considered, which has been shown to be effective in providing useful information in various domains [19].

This paper describes the method of obtaining training signals for signal classification purposes. The main idea is based on the assumption that courses of signals from a chaotic system strongly depend on initial values.

The method allows for receiving groups/clusters of signals (named refined signals) that are time-separated subsequences obtained from the source signals. Refined signals have very similar initial values (in terms of value, the first derivative and the second one).

The usage of refined signals allows for examining the influence of insignificant differences in initial conditions on a signal trajectory, which facilitates the potential detection of a chaotic property of the tested system.

The main contributions of this paper are:

- A general method of refining signals that are dedicated to chaotic property detection (https://draugustyn.gitlab.io/refined-signals-4-chaos-detection (accessed on 16 January 2025)),
- Datasets of the resulting refined signals named “Refined Datasets for Better Chaos Detection” (https://figshare.com/projects/Refined_Data_Sets_for_Better_Chaos_Detection/206641 (accessed on 16 January 2025)).
- The validation of the newly defined datasets in the classification task with the usage of a recurrent neural network.

A promising sample application of the refined signals is also provided and presented.

## 2. Materials and Methods

### 2.1. The Method of Obtaining Refined Signals

The method for obtaining the refined signals (from source data) as a chaos detection-oriented dataset is described by the following algorithm (Algorithm 1).
**Algorithm 1:** Obtaining Refined SignalsFor source input signal, reconstruct a phase portrait—obtain *M* vectors of state variables.Obtain clusters of *M*-dimensional vectors (e.g., for a 3D problem, a clustering vector is [position, velocity, acceleration]).For each cluster, select subclusters of near vectors, i.e., select the *M*-dimensional vectors closest to the center of their cluster.For each subcluster, select only those *M*-dimensional vectors that are distant enough among them in the time domain.Randomly select subclusters (containing at least two vectors). The number of selected subclusters should be large enough to satisfy the condition about the total size of samples.Generate refined signals—subsequences (substrings from the source input signal) that start from the initial vectors taken from selected subclusters.

### 2.2. Explanation of the Algorithm Steps Applied for a 3D Chaotic Dynamical Model

#### 2.2.1. Step 1—Reconstructing a Phase Portrait

During the first step of the algorithm, a one-dimensional input signal is transformed into an *M*-dimensional representation by M−1-fold numerical differentiation, giving a sequence of *M* vectors of length N−M+1. This representation of *M* vectors stands as a reconstructed phase portrait.

The order of the model is well known for dynamical systems with a given analytical definition (state equations or a differential equation). For those considered in [17], we have explicitly given M=2 or M=3.

In a general case, having no such knowledge about the model, its order and time subsequences of reconstructed state variables may be obtained by applying Takens’ Embedding method [20,21]. This allows for analyzing the system’s dynamics and estimating the order of dynamics.

This method is often used to build phase portraits and estimate the dimensionality of the system by reconstructing the phase space of a dynamical system based on one-dimensional time series data.

It involves transforming the one-dimensional data into a multi-dimensional phase space by using delayed values of the data as additional dimensions.

To illustrate the proposed method, a dataset derived from the well-known chaotic Lorenz model is taken into account. The state equations defining the Lorenz model are as follows:

$$\begin{array}{c} {\dot{x}}_{1}=-\sigma x_{1}+\sigma x_{2}\\ {\dot{x}}_{2}=\rho x_{1}-x_{2}-x_{1}x_{3}\\ {\dot{x}}_{3}=-\beta x_{3}+x_{1}x_{2} \end{array}$$

where ρ=28,σ=10,β=8/3. These equations were used to produce datasets C0ModelLorenzA51.zip (https://figshare.com/articles/dataset/C0ModelLorenzA51_zip/19919597?file=35392184 (accessed on 16 January 2025)). Values of only the first state variable $x_{1}$ taken from the mentioned datasets were used as source input signals and then used for state-space reconstruction.

**Figure 1:**
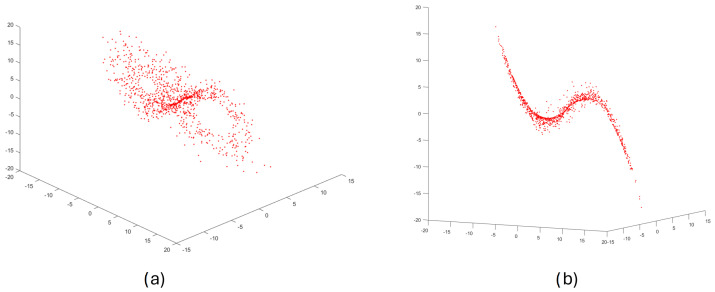
The reconstructed phase portrait for the Lorenz chaotic dynamical system ((**a**,**b**) views from different angles).

Figure 1 shows the result of performing step 1, the phase portrait for the Lorenz chaotic dynamical system in a 3D space of reconstructed state variables ($x_{1},x_{2},x_{3}$).

Although the method will be illustrated using a low-dimensional model (the three-dimensional Lorenz system), it is general in this context and can be successfully applied to high-dimensional models with a reconstructed multidimensional state-space.

#### 2.2.2. Step 2—Clustering *M*-Dimensional Data

**Figure 2:**
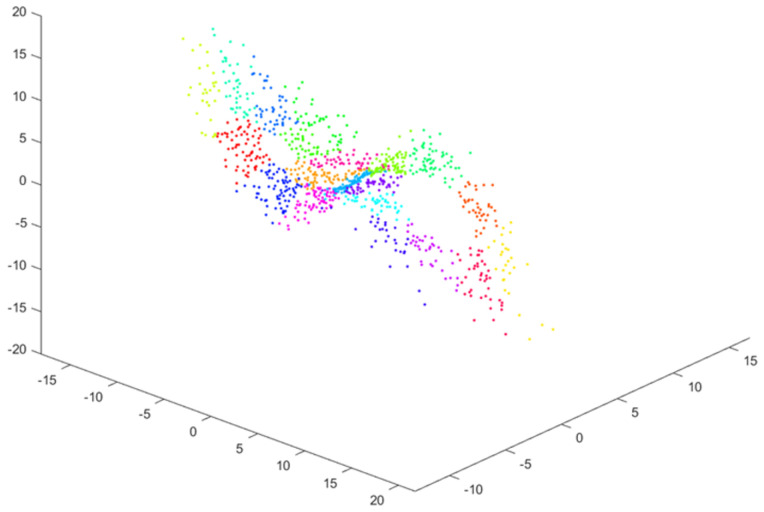
The clustering result of samples from the reconstructed phase portrait (C=20 clusters; different colors reflect belonging to different clusters).

The second step of Algorithm 1 relies on creating clusters containing vectors of the state variables that are as close as possible. The vector members are a signal value, its derivative, and its second derivative. The FCM (Fuzzy C-Means) (https://uk.mathworks.com/help/fuzzy/fcm.html (accessed on 16 January 2025)) method [22,23] is the proposed solution for the clustering task where *C* denotes the number of clusters, which is assumed to be less than or equal to sqrt(N)≈30. C=20 was used in the experiments. The results of clustering state-space data for the Lorenz system are presented in Figure 2.

#### 2.2.3. Step 3—Selecting the Nearest Vectors in Clusters

**Figure 3:**
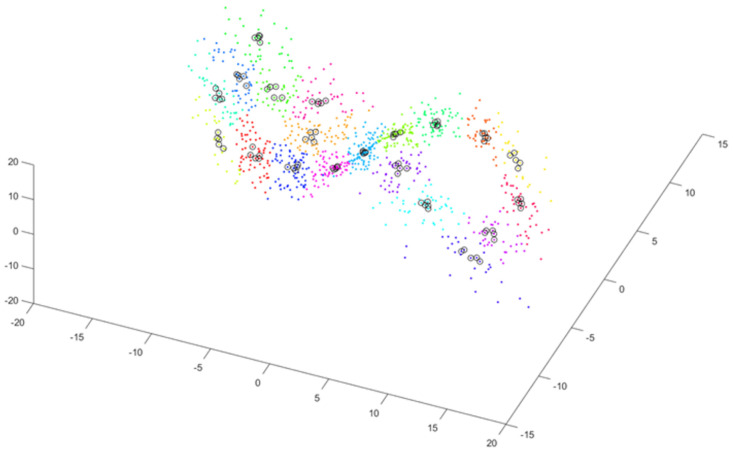
Selecting subclusters—sets of *P* vectors close to the center of a cluster (P=5)—shown by black circles.

The third step relies on determining a subcluster in each cluster that contains *P* vectors close to each other, i.e., as close as possible to the center of the cluster. Figure 3 shows subclusters of close vectors (black circles) for P=5.

#### 2.2.4. Step 4—Selecting Members of Subclusters Distant Enough in Time

**Figure 4:**
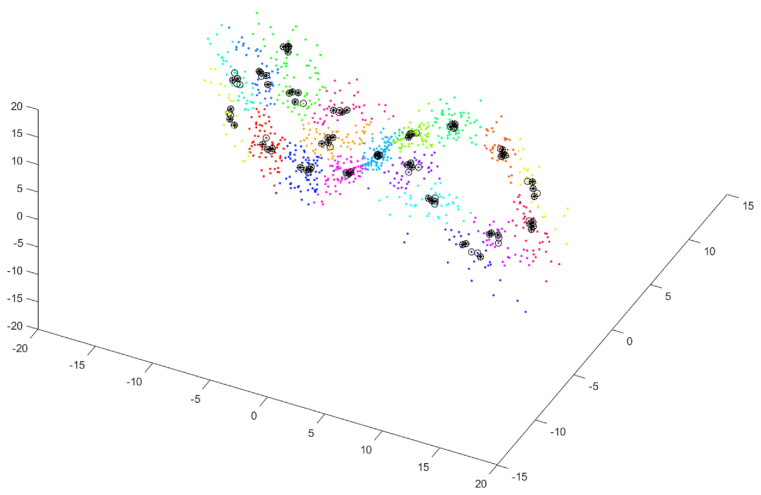
Selected vectors close to each other in terms of value but distant in the time domain for each subcluster—shown by black circles with black asterisks.

During the fourth step, for each subcluster, we select $R_{c}$ vectors from among *P* vectors $\left(0\leq R_{c}\leq P\right)$, which are separated by at least T=100 in the time domain, for c=1…C. Figure 4 shows vectors close to each other in terms of value but distant in the time domain (black circles with black asterisks).

#### 2.2.5. Step 5—Random Selecting Subclusters with Enough Size

The fifth step relies on a random selection of *W* subclusters among the ones that are large enough. $R_{w}$, the number of elements in the *w*-th subcluster (w=1…W), should be $R_{w}\geq 2$ (i.e., a subcluster has at least two subsequences with similar initial conditions that are distant along the input data series).

The number of selected subclusters should be small enough that the sum of samples $R_{w}$ multiplied by the length of subsequences will be no less than the length of the input data series, i.e., $\sum_{w}\left(R_{w}\right)\times T\geq N$.

This step relies on obtaining $\sum_{w}\left(R_{w}\right)$ subsequences whose total length is comparable to *N*. The subsequences have properties that allow for testing a dynamical system’s sensitivity to very small differences in initial conditions.

**Figure 5:**
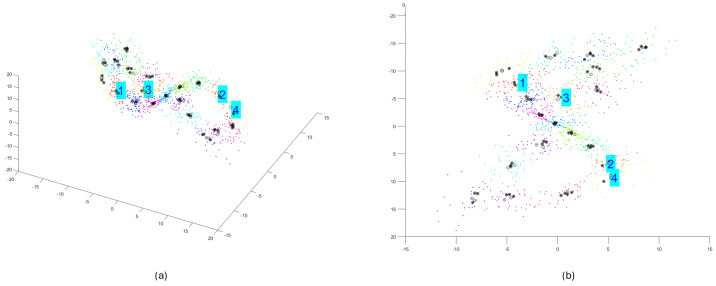
Selected four subclusters satisfying conditions of either a small distance in space or a long enough distance in time ((**a**) 3D view, (**b**) projection of the phase space on $x_{1}\times x_{2}$ plane).

Figure 5 presents randomly chosen subclusters w∈{1,2,3,4} satisfying the condition of a small distance between initial conditions in the phase space and a long enough distance in time between subsequence beginnings.

#### 2.2.6. Step 6—Generating Refined Subsequences with Enough Size

**Figure 6:**
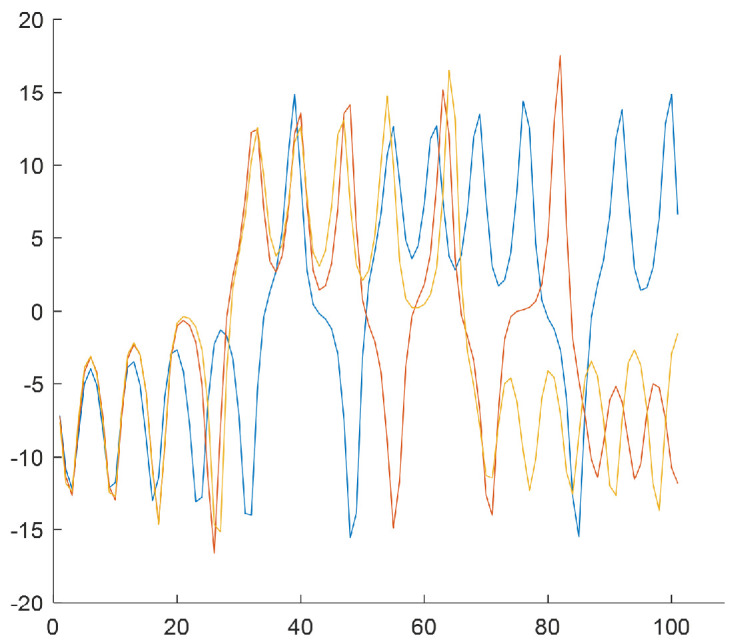
$R_{w}=3$ subsequences for the w=1 (the first subcluster). Center of cluster no. w=1 in the phase space: [−7.5860,−3.7564,3.1256]. Time shifts of the beginning of subsequences: 76, 277, 657.

**Figure 7:**
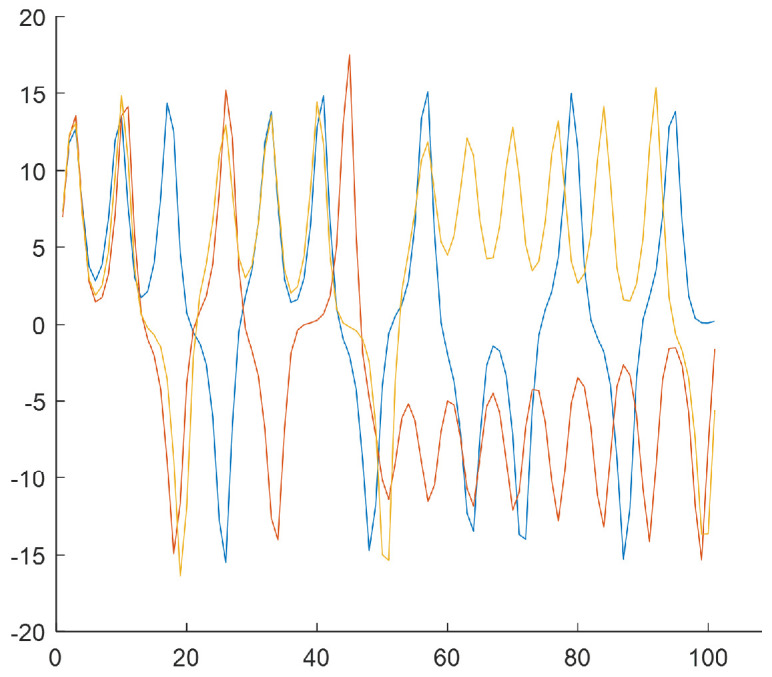
$R_{w}$ = 3 subsequences for the w=2 (the second subcluster). Center of cluster no. w=2 in the phase space: [7.0294,4.9233,−3.6932]. Time shifts of the beginning of subsequences: 135, 314, 470.

**Figure 8:**
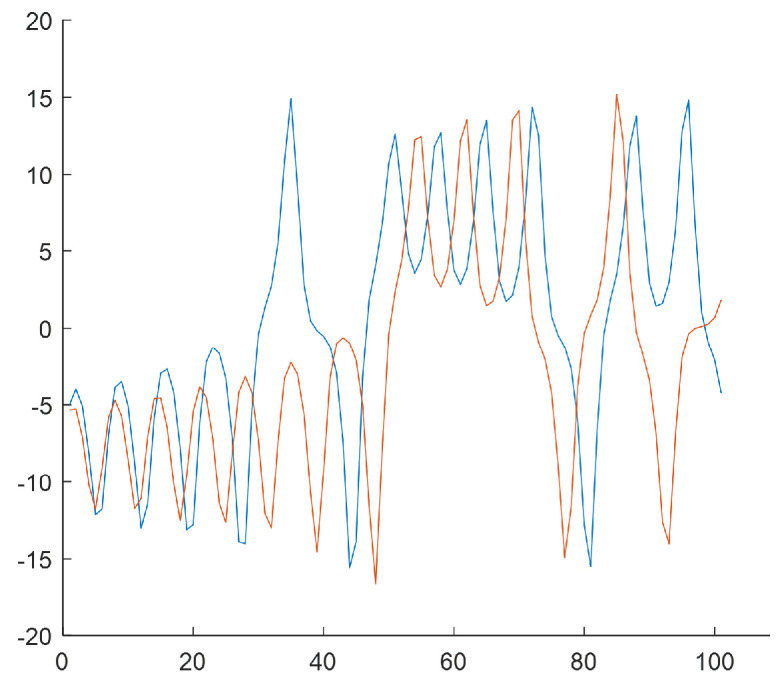
$R_{w}=2$ subsequences for the w=3 (the third subcluster). Center of cluster no. w=3 in the phase space: [−4.9350,0.5319,−1.8641]. Time shifts of the beginning of subsequences: 80, 255.

**Figure 9:**
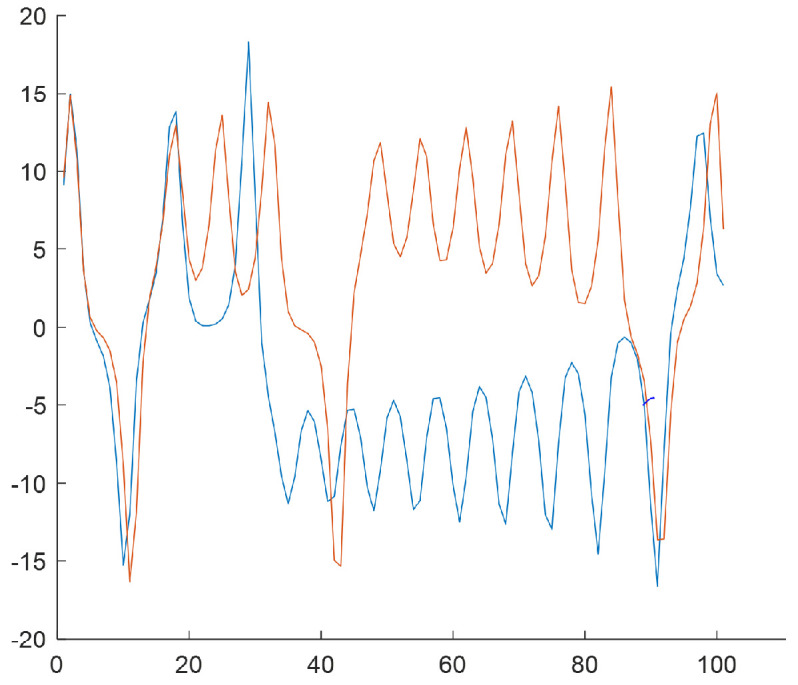
$R_{w}=2$ subsequences for the w=4 (the fourth subcluster). Center of cluster no. w=4 in the phase space: [9.4861,5.2908,−9.2462]. Time shifts of the beginning of subsequences: 212, 478.

The last step of the algorithm relies on generating subsequences of length T=100 based on *W* subclusters by selecting samples from the *N*-element input data series. The subsequences start from the values that result from subclusters w∈{1,2,3,4} Figure 6, Figure 7, Figure 8 and Figure 9 show the final result of the method, i.e., subsequences obtained for each subcluster.

Because of the chaotic nature of the Lorenz system, the obtained subsequences significantly differ even if they begin from a similar initial condition. The figures show time courses of signals belonging to the same the *w*-the subcluster. The figures confirm the sensitivity to minimal differences in initial conditions. Each of the obtained refined datasets consists of subsequences with initial conditions belonging to the *w*-th subcluster. These initial conditions are close to the center of a subcluster and the vectors of subcluster centers are presented in Figure 6, Figure 7, Figure 8 and Figure 9. Each subsequence (with a length equals 100) is taken from an input sequence (with a length equal to 1000). Locations of the beginnings of subsequences within the input sequence are called time shifts and their values are presented in Figure 6, Figure 7, Figure 8 and Figure 9 too.

The result is $R_{1}+R_{2}+R_{3}+R_{4}=3+3+2+2=10$ subsequences, each of them of the length T=100. This provides 10×100=1000 samples, which is equal to the length of the input time series (*N*).

### 2.3. Example of Applying the Method for Chaotic Dynamical Model and Non-Chaotic One

To illustrate the proposed method in the context of different types of systems, two dynamical models [17] were used—the chaotic Rössler model and the non-chaotic Linear Oscillator model.

**Figure 10:**
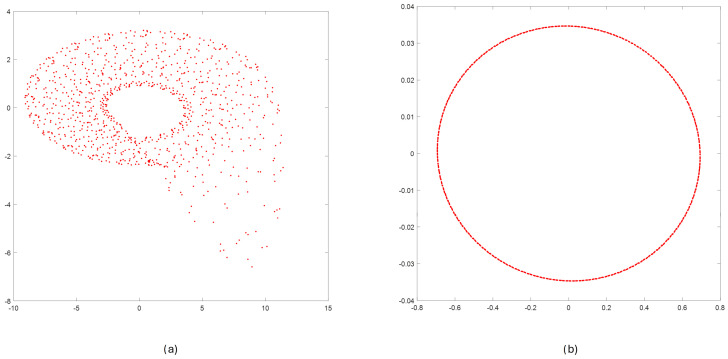
Result of step 1—reconstructing values of the state vector. Two-dimensional projection of phase space for Rössler (**a**) and Linear Oscillator (**b**).

After step 1, we obtained reconstructed phase portraits in 3D and 2D, respectively. To provide a uniform view for both models, the 2D projections of the phase portraits (values of signal and its first derivative) are presented in Figure 10.

**Figure 11:**
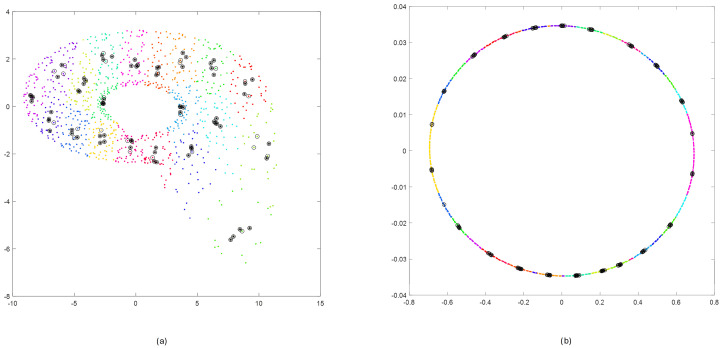
Results of steps 2, 3, 4 for reconstructed phase portraits of Rössler (**a**) and Linear Oscillator (**b**): clustering (step 2)—colored points; selecting the nearest points inside a cluster (step 3)—black circles; selecting points distant in time by more than T=100 (step 4)—circles with an asterisk.

The effects of applying steps 2, 3, and 4 for reconstructed phase portraits for Rössler and Linear Oscillator are shown in Figure 11.

**Figure 12:**
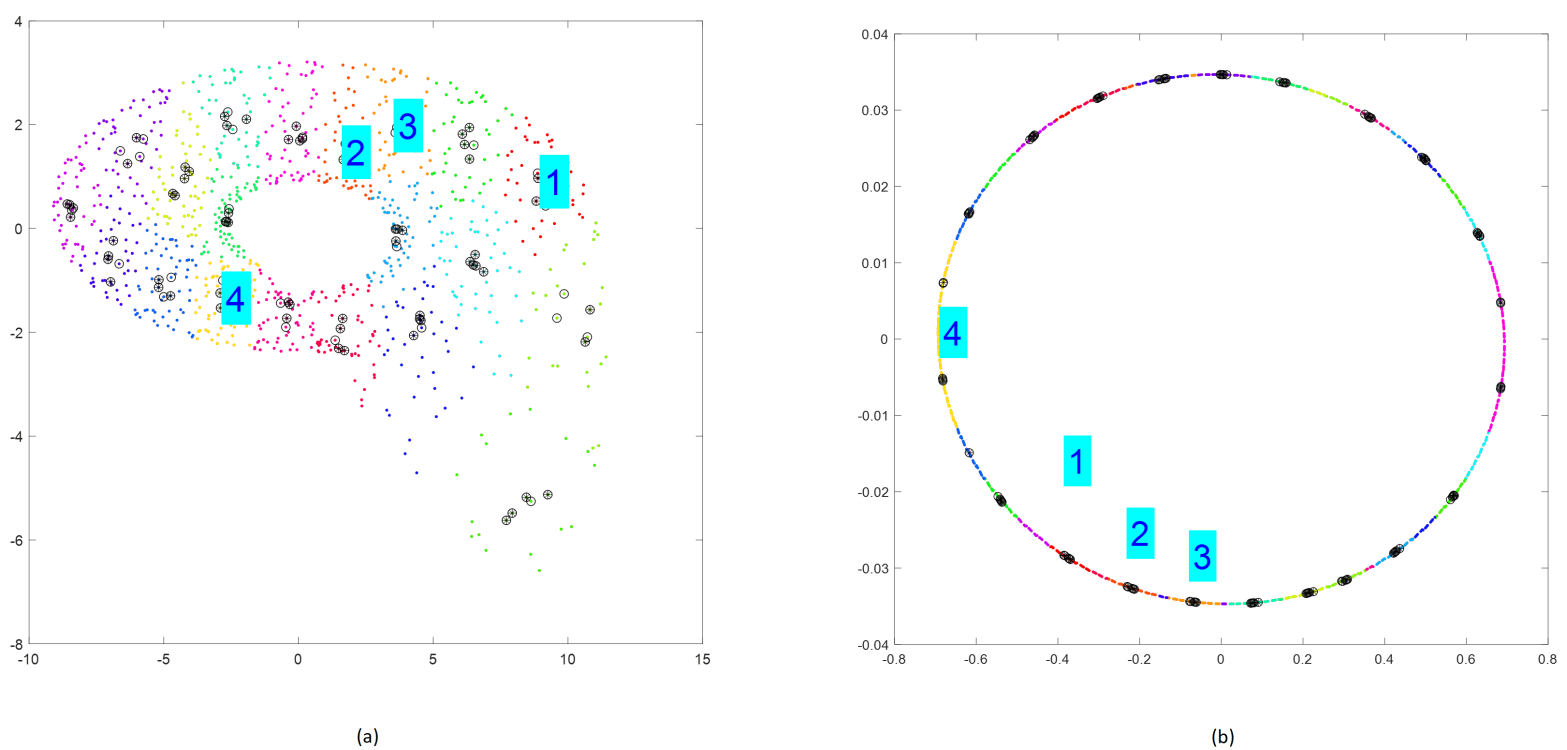
Selection of four subclusters by executing step 5 of Algorithm 1 applied for Rössler (**a**) and Linear Oscillator (**b**).

Randomly selected clusters after performing step 5 are presented in Figure 12.

**Figure 13:**
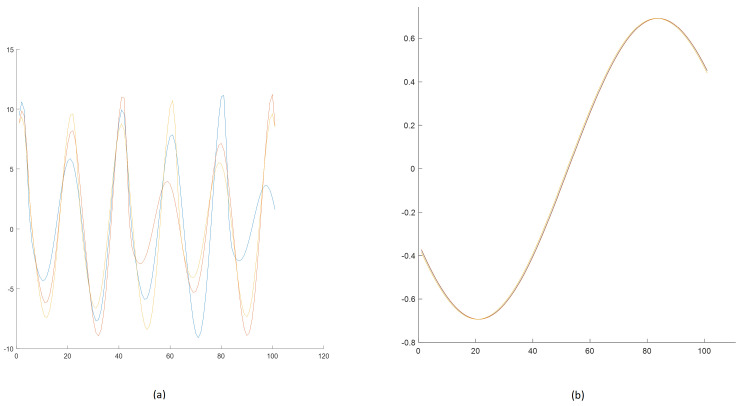
Subsequences with very similar initial conditions—three subsequences per subcluster: (**a**) subsequences for subcluster no. 1 selected from Figure 12a (Rössler), (**b**) subsequences for cluster no. 2 selected from Figure 12b (Linear Oscillator).

Figure 13 presents the results of step 6. It shows the final subsequences for cluster no. 1 for Rössler and cluster no. 2 for Linear Oscillator.

Three subsequences in Figure 13 significantly differ regarding the ending values (near 100) compared to those in Figure 13b. Of course, this was expected due to the chaotic properties of the Rössler model.

**Figure 14:**
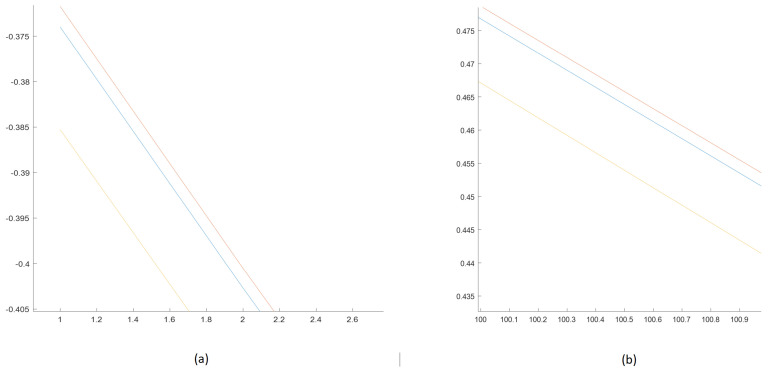
Zoomed-in parts of Figure 13b—beginning (**a**) and ending (**b**) of the subsequences for Linear Oscillator (distances between the signals are constant).

To show that the initial small differences among the subsequences remain small for a non-chaotic system, Figure 14 presents zoomed-in courses of subsequences for the initial (a) and final (b) moments of time.

## 3. Results

### 3.1. Refined Datasets for Better Chaos Detection

The general goal of this work is to present the proposed method (defined in Section 2.1 and illustrated in Section 2.2) for obtaining refined signals that may support the detection of chaotic behavior. This method allows the extraction of refined signals (subsequences) from some source 1D signals (input sequences).

The direct result of the presented research is the prepared datasets, which are accessible via the project “Refined Datasets for Better Chaos Detection” (https://figshare.com/projects/Refined_Data_Sets_for_Better_Chaos_Detection/206641 (accessed on 16 January 2025)). The project consists of signal datasets for selected dynamical models (chaotic and non-chaotic).

Each dataset consists of 10 subsets, including the following:

- Refined signals—a few (2–4) subsequences (each with a length equal to 100 samples) belonging to a subcluster, with a similar initial condition;
- A source signal—an input sequence with a length equal to 1000 samples.

Most of the prepared refined datasets come from synthetic signals derived from the simulation of mathematical models (both chaotic and non-chaotic ones). However, the proposed refining method is general and can also be applied to refining real signals derived from sensors. Thus, additionally, to present the usage of sensor-based datasets, we considered an input signal that was obtained using the photoplethysmography (PPG) technique. It comes from the PPG-DaLiA dataset (https://ubicomp.eti.uni-siegen.de/home/datasets/sensors19/ (accessed on 16 January 2025)), which contains recordings made using the Empatica E4 wristband (Empatica Inc., Cambridge, MA, USA) for a range of activities of daily living. In addition to the photoplethysmography sensor, this device has three other sensors: an electrodermal activity sensor, a three-axis accelerometer, and an optical thermometer. These sensors register the following data: blood volume pulse, inter-beat interval, electrodermal activity, XYZ raw acceleration, and skin temperature.

For the purpose of applying the proposed method for sensor data, the input PPG dataset, which contains the input sequence (with 10,000 samples), was taken from PPG_FieldStudy\S3\S3_E4.zip\BVP.csv. After processing the PPG signal using the proposed refining method, we obtained 30 new refined dataset files (with 1000 samples) that were enabled as a figshare resource (https://figshare.com/articles/dataset/Data-PPG-BVP-refined_-_source_signals_refined_ones/28233026 (accessed on 16 January 2025)). The illustration of results after executing the steps of the method while obtaining the PPG refined dataset was enabled via GitLab repository (https://gitlab.com/draugustyn/refined-signals-4-chaos-detection/-/blob/main/PPG-dataset-refining.pdf (accessed on 16 January 2025)).

### 3.2. Sample Usage of Refined Data for Improving Classification

The data obtained with the previously described method were verified in terms of their usefulness in the process of distinguishing chaotic and non-chaotic signals. For this purpose, an experiment was conducted in which models were trained using three datasets. The first one contained the new data presented in this paper, further referred to as the Refined dataset. The second set contained the time series of the source signals that [17] used to create the Refined dataset—referred to as the Original dataset. The third dataset was obtained by augmenting the Original dataset with samples from the Refined dataset. Time series with *n* elements (n=100) were defined for each dataset. The models’ validation was realized utilizing the test dataset described in [17], divided into *n*-element time series. Table 1 includes the number of time series in each dataset.

The experiment was conducted using a computer with an Intel(R) Core(TM) i9-10900F CPU @ 2.80 GHz processor and 32.0 GB RAM. The computer had a 64-bit operating system installed.

As mentioned, the main goal of the experiment was to verify if the models trained on the newly created datasets could perform better compared to models trained on the Original one [17]. Therefore, a very simple LSTM neural network consisting of an input layer, one hidden layer with *U* units, and a dense layer with the SoftMax activation function was utilized. The models were trained with the Categorical Cross-Entropy as the loss function and the Adam optimizer with standard parameters (learning rate equal to 0.001). As presented in [24], the Adam optimizer, when used for neural networks, made faster progress, required little memory, and showed better convergence than other methods. The experiment was conducted three times with different numbers of units in the hidden layer: U={4,16,64}. The training phase lasted 1000 epochs with BATCH_SIZE =64.

**Figure 15:**
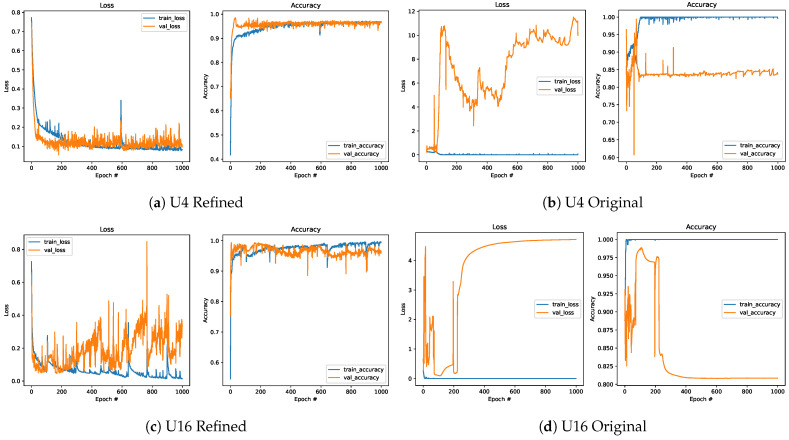

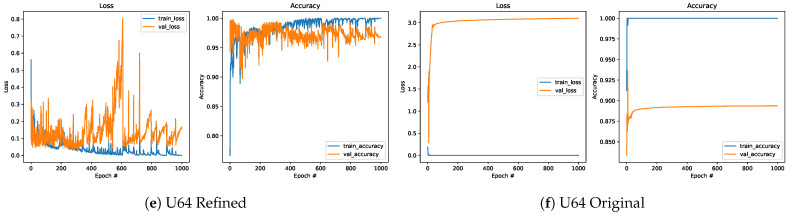
Comparison of the loss function and accuracy for the Refined (**left**) and Original (**right**) datasets.

When the obtained results for the models defined based on the Refined (Figure 15a,c,e) and Original datasets (Figure 15b,d,f) are collated, it can be noted that models trained on data from the new datasets are more efficient than those for the other dataset, despite having a lower number of training samples. This was observed even when the simplest network with U=4 is considered. The Refined dataset achieved better performance (approximately equal to or above 95%) than the Original one (equal to approximately 84%). For a higher number of units, models trained on the Refined dataset kept performing well, with accuracy reaching approximately 96–98%, while for the models defined based on the Original dataset, an accuracy plateau of approximately 82–90% was reached.

**Figure 16:**
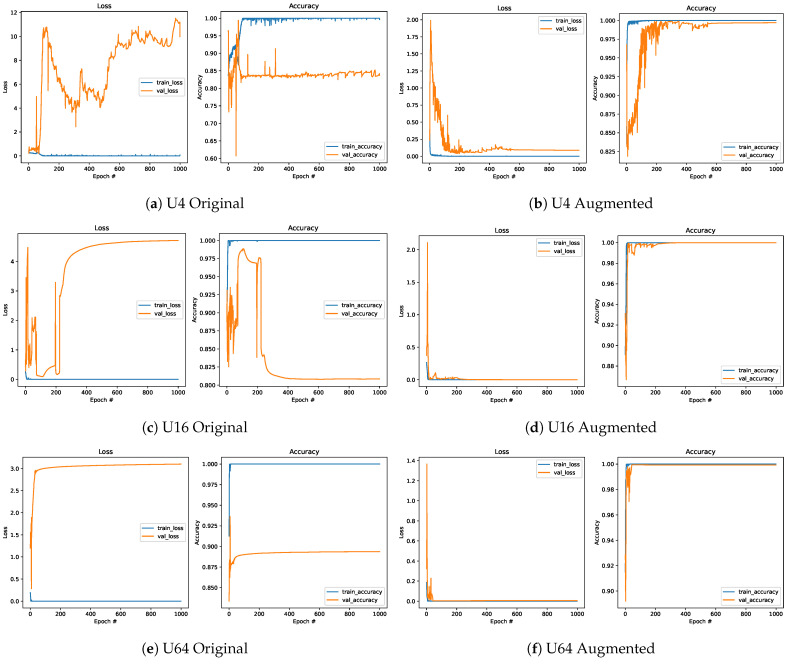
Comparison of the loss function and accuracy for the Original (**left**) and Augmented (**right**) datasets.

The usability of the new approach for defining the training dataset was also confirmed by comparing the models trained on the Original (Figure 16a,c,e) and Augmented datasets (Figure 16b,d,f). It can be noted that adding Refined data series to the Original dataset significantly improved the model’s performance. A slight improvement is also visible compared to the models trained on the Refined dataset. However, the model trained on the Augmented dataset is five times larger than the model defined on the Refined dataset.

## 4. Discussion and Conclusions

The approach presented in this study aimed to develop a method for generating new signal datasets designated for chaos detection. This was achieved by focusing on generating signals with similar initial conditions from some other general signals.

The first direction of future work will concentrate on improving the proposed method of generating refined datasets.

The proposed method will be extended and verified for the use of other state-space clustering methods (other than FCM proposed in Section 2.1—step 2).

Another extension of the method, namely the use of different subcluster selection methods (other than the simple random one proposed in Section 2.1—step 5), will be explored, too. The goal is to select subclusters that are compact (and are large enough) but as distant as possible (in the sense of mutually distant centers of these subclusters). This will allow for better distribution of initial conditions of generated subsequences in the sense of fully covering the entire reconstructed phase space (in contrast to random selection).

The second direction of future work will concentrate on applying the refined datasets in different ways for signal classification.

The datasets were meant to improve model training and its performance in distinguishing signal types, and turned out to be an effective solution. They not only shortened the training phase but also ensured better results. The times series generated using the new method were used to train the simple LSTM recurrent network in various configurations. The efficiency of the obtained models was promising. Their accuracy in differentiating chaotic and non-chaotic behavior compared to that achieved for the Original dataset was improved from approximately 84% to approximately 95%. Further improvement were obtained by merging samples from the Refined and Original datasets. The accuracy reached even 99% in this case.

However, there is still room for the development of this method.

Future application of the models trained on the Refined dataset against biological signals requires further investigation. Disorder and noise are intrinsic in biological systems. Noise is a part of the flexibility and plasticity of such systems and provides them with the advantages needed for proper functioning [25]. Therefore, subsequent experiments are planned. The efficiency of the obtained models will be checked against data, including noise. Two scenarios are planned to be considered: training models with clean and noisy data and their evaluation on a test set, including signals with added noise. Replacing the LSTM network with a convolutional one is also being considered.

Our future research will concentrate on different approaches to the verification of the usefulness of original datasets [17] and refined ones regarding the correctness of signal classification, and thus we plan to verify this by applying the hybrid method [26], based on the Chaos Decision Tree Algorithm.

The usefulness of the new datasets (the refined ones) will be verified in the future by also using classical methods of nonlinear time series analysis, i.e, those based on the following:

- The value of the largest Lyapunov exponent (positivity for chaotic systems),
- The fractal dimension (smaller than topological dimension for chaotic systems),
- KS-Entropy (positivity for chaotic systems),
- The results of statistical test, consisting of comparing the original set with the so-called surrogate data (generated data from the original set with random chaos). The method is implemented by randomizing phases in the frequency domain while maintaining the amplitude of the Fourier spectrum of the original data—the significance of the difference of Lyapunov coefficients between the original data and for the surrogate data indicates chaos.

In future work, the usability of the obtained Refined dataset for high-dimensional data will be verified, too. It is planned that these data will come from simulations of nonlinear space–time systems (e.g., models describing flow velocities, such as the Navier–Stokes model or the Kuramoto–Sivashinsky model).

## Figures and Tables

**Table 1:** Description of the datasets.

	Dataset			
Time Series	Refined	Original	Augmented	Test
All	1200	70,000	71,200	48,840
Chaotic	500	30,000	30,500	18,840
Non-chaotic	700	40,000	40,700	30,000

## Data Availability

The data that are considered in this paper are publicly available as part of a project enabled by the figshare website (https://figshare.com/projects/Refined_Data_Sets_for_Better_Chaos_Detection/206641 (accessed on 16 January 2025)).
